# Changes in Secondary Metabolites Content and Antioxidant Enzymes Activity in Leaves of Two *Prunus avium* L. Genotypes During Various Phenological Phases

**DOI:** 10.3390/life14121567

**Published:** 2024-11-29

**Authors:** Jan Kubes, Frantisek Hnilicka, Pavla Vachova, Jiri Kudrna, Barbora Tunklova, Miloslav Mrkacek, Tomas Rygl

**Affiliations:** Department of Botany and Plant Physiology, Faculty of Agrobiology, Food and Natural Resources, Czech University of Life Sciences Prague, Kamýcká 129, 165 00 Praha-Suchdol, Czech Republic; hnilicka@af.czu.cz (F.H.); vachovap@af.czu.cz (P.V.); kudrnaj@af.czu.cz (J.K.); tunklova@af.czu.cz (B.T.); ryglt@af.czu.cz (T.R.)

**Keywords:** sweet cherry, phenology, abiotic stress, secondary metabolites, enzymatic activity

## Abstract

In addition to its fruit, the sweet cherry (*Prunus avium* L.) has other parts that can be used as a source of compounds with beneficial biological activity. The content of these metabolites is affected by different inner and outer factors, often as a response to plant defense against various stresses. Leaves of two *P*. *avium*. genotypes, Kordia and Regina, grafted on the same rootstock, were analyzed from trees grown in orchards in six different phenological phases for two years. The content of several groups of phenolic compounds, lipid peroxidation, antioxidant activity of the extracts, and enzyme activity were observed via colorimetric methods on a UV/Vis spectrophotometer. The obtained data showed that the content of metabolites and other parameters in these two genotypes are dependent on the term of harvest, as well as environmental conditions, mainly temperature, but sunshine duration and rainfall also had a certain effect on the compounds in the leaves of Kordia and Regina. Even though the differences between these genotypes were not always significant, it is important to consider the right time to harvest the leaves of the sweet cherry, as their content could vary as a result of the reaction to various other conditions and could reflect the resistance of the chosen genotype.

## 1. Introduction

The sweet cherry (*Prunus avium* L.), a member of the Rosaceae family, represents a significant source of widely consumed fruits. In the Czech Republic, its cultivation currently spans an area of 7 km^2^ (700 ha) [1]. The fruits of *P. avium* are notable for their abundance of health-promoting compounds, including vitamins, dietary fiber, and polyphenolic metabolites. Among these metabolites are glycosides of flavonoids [2,3], such as flavonols (quercetin, kaempferol), flavanols (catechin, epicatechin), and phenolic acids (chlorogenic, neochlorogenic, and structurally related or derived acids). These bioactive compounds are primarily responsible for the antioxidant properties of sweet cherry fruits [4]. The geographical origin of *P. avium* remains a subject of debate, with some researchers tracing it to Asia [5], while others suggest a European origin [6], based on archaeological and fossil evidence.

The entire genus *Prunus*, not only *P. avium*, holds significant importance for horticulturists and fruit producers, encompassing approximately 77 known species. Due to its polymorphism and the existence of numerous distinct genotypes, the taxonomy of this group remains complex [7]. As a mesophyte, sweet cherry requires soil with adequate water content, as excessively dry or humid conditions can negatively impact fruit production [8]. Additionally, *P. avium* thrives in warmer climates with ample sunlight. Grafting and the selection of appropriate rootstocks, whether generative or vegetative, are crucial for enhancing vitality and achieving higher yields, particularly in suboptimal environmental conditions. According to [9], the chosen rootstock genotype influences various physiological processes, including chlorophyll fluorescence, gas exchange, and water management, as also demonstrated by [3], who highlighted effects on different plant organs and secondary metabolite content. As noted, the primary purpose of *P. avium* cultivation is its fruit, valued not only for its nutritional properties but also for its array of health-beneficial compounds. Recently, several studies [10,11,12] and reviews [2,13,14] have explored the potential use of other plant parts and their extracts as alternative sources of biologically active compounds. Methanolic extracts from leaves have demonstrated in vitro inhibitory effects on α-glucosidase and α-amylase (antidiabetic activity) as well as tyrosinase, acetylcholinesterase, and butyrylcholinesterase (anti-Alzheimer’s activity) [11,15].

The fruits and leaves of *P. avium* have demonstrated positive effects on lipid metabolism in rats fed a high-cholesterol diet [10] and exhibit anti-hyperglycemic activity [2,16]. By-products of *P. avium* may thus have potential applications in the treatment of diseases associated with cardiovascular and metabolic systems or in alleviating related complications. Leaves have also been evaluated for their anti-inflammatory and antimicrobial properties [17], although studies often report material collected only during specific intervals of a single season, if detailed at all [18].

Compared to fruits, *P. avium* leaves have been found to contain higher levels of carotenoids, ascorbic acid, polyphenols, and other bioactive compounds. Higher antioxidant activity, linked to these compounds and phenolic acids, has also been reported [13,19]. However, the genus *Prunus* is well known for the presence of cyanogenic glycosides, whose concentrations vary throughout the year. These compounds, such as prunasin, may exert toxic effects, though they have also been explored for their therapeutic potential in treating various symptoms and illnesses [13].

Environmental conditions significantly influence the quality and quantity of metabolites not only in fruits [20], but also in other plant parts [21]. Factors such as fruit ripening stage, cultivated genotype, and organ position on the tree [22] can affect metabolite content. Similarly, the metabolite profile of *P. avium* leaves is also subject to these variables [23]. In cases of harvest loss due to unfavorable environmental conditions (e.g., spring frost or rain during harvest causing fruit rupture), the collection of leaves at appropriate developmental stages could serve as an alternative source of production, potentially mitigating economic losses for producers [24,25]. Additional factors, such as rootstock selection and bud development timing, may provide some protection against these adverse effects [26].

Since differences in fruit characteristics between the Kordia and Regina genotypes on the Gisela 5 rootstock have been documented [27], this study focused on changes in various biochemical parameters in their leaves, examining the impact of different phenological phases and environmental conditions over two consecutive years.

## 2. Materials and Methods

### 2.1. Orchard Growing Conditions

Plant leaf material was acquired from trees cultivated in Research and Breeding Fruit Institute Holovousy Ltd. (RBFI) in Holovousy, Czech Republic, localized in the northeast of Bohemia (50.37608 N, 15.58143 S; altitude 360 m). The region is characterized by a warm and mildly humid climate with brown earth soil, and the orchard is orientated toward the south. The trees were planted in 2008 without additional irrigation and netting systems. For the comparison of biochemical traits, two genotypes were chosen—Kordia and Regina. The rootstock Gisela 5 (*Prunus cerasus* × *Prunus canescens* [27]) was used for both variants, and the trees were planted in 5 × 1.5 m spacing. The plants were treated by standard agrotechnological methods and guidelines of the Association for Integrated Systems of Fruit Growing CR. As fertilizer was used LAV 27 (ammonium nitrate + limestone) in dose 110 kg/0.01 km^2^ (1 ha) once per year, with additional fertilizers (DAM 390 and STOPIT) applied to the leaves during the year and fruits ripening.

### 2.2. Harvesting of Plant Material

**Figure 1:**
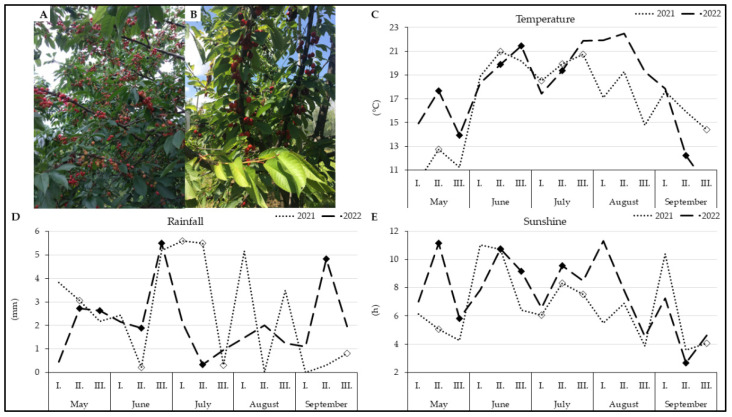
Sweet cherry genotypes and climatic data with terms of harvest (BBCH 65–92), marked by diamonds, during season 2021 (dot lines and white diamonds) and 2022 (dash lines and black diamonds): (**A**) Kordia; (**B**) Regina; (**C**) Temperature; (**D**) Rainfall; (**E**) Sunshine. Roman numbers mark the third of each month.

The leaves were harvested from selected trees in six terms (Table S1) according to phenological phases (BBCH—Biologische Bundesanstalt, Bundessortenamt and Chemical industry) during 2021 and 2022. Phases were same both years, but the dates were slight shifted as result of different climatic conditions on beginning of 2022. This is also shown in Figure 1C–E by diamonds mark on the curves. Progress of particular stages were observed and communicated by Ing. Suran from RBFI and the phases were selected according to literature [28]. The leaves were packed in aluminium foil and transported in cooled conditions to laboratory of Department of Botany and Plant Physiology and immediately analysed or stored in −80 °C before further use.

### 2.3. Extract Preparation and Estimation of Malondialdehyde

Membrane damage, as result of lipids degradation, was estimated by method of [29]. Briefly, 0.5 g of leaves were homogenized in liquid N_2_ and extracted with 10.5 mL of 80% (*v*/*v*) ethanol. Same volumes of extract and reagent containing 2-thiobarbituric acid in trichloroacetic acid with butylated hydroxytoluene were mixed up and heated in glass test tubes in waterbath (95 °C; WNB22; Memmert; Büchenbach, Germany) for 25 min. Supernatant for spectrophotometric analysis was prepared by centrifugation (Frontier 5718R; Ohaus; Nänikon, Switzerland) of samples in 12,700 rcf (relative centrifugation force) for 1 min. An absorption was taken against distilled water as blank at 440, 532 and 660 nm on UV/Vis spectrophotometer (Evolution 201; Thermo Scientific; Waltham, MA, USA), and acquired values were used for calculation of malondialdehyde (MDA) content in nmol/g of fresh weight (FW; [29]).

### 2.4. Estimation of Secondary Metabolites and Antioxidant Capacity

For the determination of total phenolic content (TPC), the method of [30] was used. Briefly, aliquot of ethanolic extract from MDA analysis was mixed with ten times diluted Folin-Ciocalteu’s reagent and 7% Na_2_CO_3_ and the absorbance of sample was taken at 760 nm after 90 min. TPC was estimated in mg of gallic acid equivalents (GAE)/g of fresh weight (FW) used for the calibration curve (Figure S1A).

Adapted method of [31] was used for determination of total flavonoid content (TFC) in same ethanolic samples of cherry leaves. The extracts were incubated with 5% NaNO_2_ for 5 min, 10% AlCl_3_ for 6 min and the mixture absorbance was measured at 415 nm after adding of 1M NaOH. Between each step, reaction mixtures were vigorously shaken. TFC was calculated as mg of quercetin equivalents (QE)/g FW (Figure S1B) in this analysis.

Total phenolic acids content (PAC) was determined according to the assay of [32], when the ethanolic extract was combined with 0.5 M HCl, 10% NaNO_2_, 10% NaMoO_4_ and 1 M NaOH. An absorbance of mixture was measured at 490 nm and a caffeic acid was used as standard for the calibration curve (Figure S1C). The values are in mg of caffeic acid equivalents (CAE)/g FW.

Final method using extract from MDA is adapted from [33]. The aliquot of sample was mixed with reagent solution (H_2_SO_4_, KH_2_PO_3_, NaMoO_4_) and heated in 95 °C for 90 min. After cooling, absorbance of solution was taken at 695 nm and total antioxidant capacity total antioxidant capacity was calculated as mg of ascorbic acid equivalents (AAE)/g FW (Figure S1D).

### 2.5. Estimation of Antioxidant Enzymes

The enzyme extract was prepared by homogenization of 0.3 g of leaves, powdered in liquid N_2_, in 3 mL of phosphate buffer (50 mM, pH 7.0) containing polyvinylpyrrolidone and EDTA. The suspension was centrifuged at 20,600 rcf, 4 °C for 20 min. Aliquot of supernatant was mixed with Bradford solution and content of proteins was measured at 750 nm with a bovine serum albumin as standard for the calibration curve.

Activity of catalase (CAT; [34]), guaicol peroxidase (GP; [35]) ascorbate peroxidase (ASX; [36]) and superoxide dismutase (SOD; [37]) was estimated according to respective authors with slight modifications [38] and expressed as U/min/mg of proteins. All enzymatic analyses were performed in 96-plate microplates and measured on multiplate reader (Tekan Spark, TECAN; Mannedorf, Switzerland).

### 2.6. Statistical Analysis

A factorial analysis of variance (ANOVA) was employed for the purpose of comparing the measured variables. Following the attainment of significant results (*p* < 0.05), multiple comparisons were conducted using Tukey’s Honestly Significant Difference (HSD) test in order to identify significant differences between the various phenological phases and/or genotypes. In case of present significant difference between values, different letter were used for highlighting them.

All analyses were conducted using the statistical software package Statistica ver13.5 (Statsoft, Tulsa, OK, USA). Redundancy analysis (RDA) was performed using the Canoco 5 software [39]. This analysis was employed to visualise the differences in phenological phases between the two years under consideration, with years and genotype used as supplementary variables and precipitation, sunshine and temperature were used as explanatory variables.

All measurements were made as means (M) with standard deviations (SD) from at least six repetitions. Meteorological data was taken as mean of values from at least ten days before leaves harvesting.

## 3. Results

### 3.1. Climatic Data

All climatic data were obtained from the website of the Czech Hydrometeorological Institute [40], which has a station for measuring observed parameters in RBFI Holovousy area. The progress of air temperature (Figure 1C) had been similar during both years, but the values were higher in 2022, especially in spring and August. However, the drop of temperature was more abrupt in September of the second year.

Regarding precipitation, there were fluctuations in some observed stages between both years. Generally, it could be seen (Figure 1C) that September 2021 had less of rainfall, but this year had been rainier during the rest of set out seasons with some exceptions like beginning of April middle of June or the end of July. The last meteorological parameter, sunshine duration (Figure 1E), represents the mean hours of sunshine with an intensity above 120 W/m^2^ during the observed period. In summary, sweet cherry leaves were exposed to more sunlight in 2022 than in the previous year, exception for early June, September and late April. This is also in presumed correlation with previous parameters as the year 2021 was colder and rainier (Figure S2).

### 3.2. MDA Content

**Figure 2:**
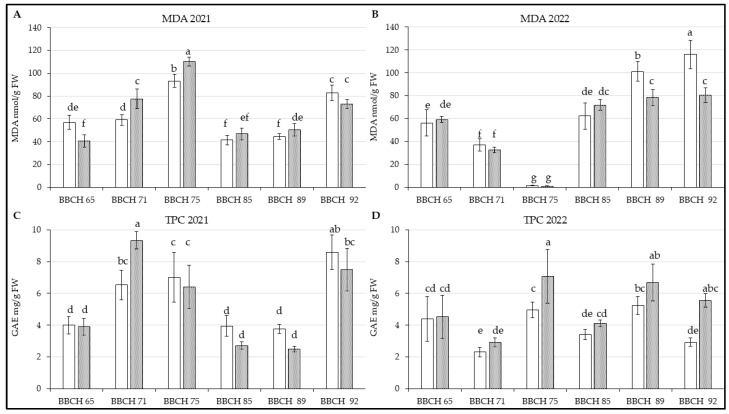
Content of measured parameters in leaves of *P. avium* two genotypes: (**A**) MDA in 2021; (**B**) MDA in 2022; (**C**) TPC in 2021; (**D**) TPC in 2022. Results are mean of 6 values ± SD. Factorial ANOVA was made for evaluation of differences if *p* < 0.05 with Tukey’s post-hoc test. Various letters show significant differences between values of genotypes Kordia and Regina in six BBCHs in observed years. The Y-axes show the measured parameters, and the X-axes show the phenological phases (BBCH65-BBCH92) for both genotypes.

As shown in Figure 2A [F(5, 66) = 21.447; *p* < 0.01], the content of malondialdehyde increased in the leaves of both genotypes during the first three stages of observation in 2021, peaking at BBCH 75, when the 50% of fruits on the trees reached their final size or the fruit measured 50% of its final size (Table S1). During the rest of summer, the values had been lower and relatively similar, but in September, amount of MDA increased again. In most observed intervals, higher MDA content was recorded in Regina compared to the other genotype, except during the first interval, where the difference between them was statistically significant, and the last one.

In the following year [Figure 2B; F(5, 66) = 16.758; *p* < 0.01], the progress of MDA content in sweet cherry leaves was rather different; it had decreased during the first three stages of harvest, and it was lowest at BBCH 75 at all. However, similar drop in leaves MDA content was observed in the case of other species like *Prunus armeniaca* L. growing in same site (unpublished data) in same year. Subsequently, MDA levels gradually increased, particularly in Kordia. A statistically significant difference in MDA content between Kordia and Regina was observed only in the last two stages, with their concentrations being more similar compared to 2021.

### 3.3. Secondary Metabolites Content and Antioxidant Capacity

#### 3.3.1. Total Phenolic Content

As with MDA, total phenolic content increased during the first three harvests of the 2021 season, showing a similar overall trend for both genotypes in the course of the observed phenological stages [Figure 2C; F(5, 66) = 9.6679; *p* < 0.01];. However, significantly more of TPC was measured in the leaves of Regina in BBCH 71. In the rest of this year, TPC was higher more likely in samples from Kordia, but the results did not show significant differences, and the visible changes are rather between some phenological stages.

In 2022 [Figure 2D; F(5, 66) = 4.1432; *p* < 0.01], it could be seen that genotype Regina had higher TPC in leaves in comparison with Kordia, significant especially in BBCH 75 and BBCH 92. Although several fluctuations in TPC were recorded throughout the year, the general trend was similar for both genotypes.

#### 3.3.2. Total Flavonoid Content

**Figure 3:**
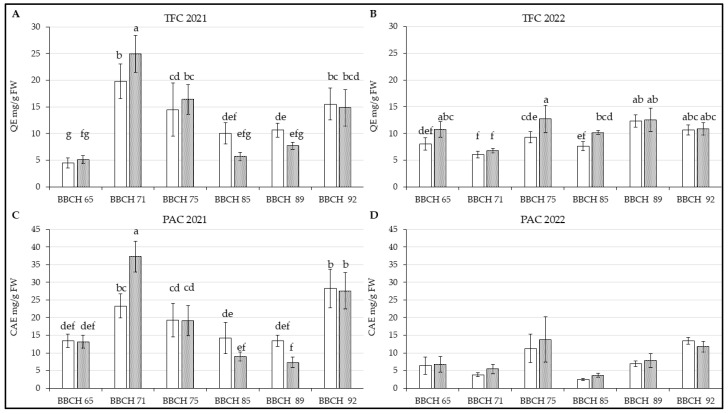
Content of measured parameters in leaves of *P. avium* two genotypes: (**A**) TFC in 2021; (**B**) TFC in 2022; (**C**) PAC in 2021; (**D**) PAC in 2022. Results are mean of 6 values ± SD. Factorial ANOVA was made for evaluation of differences if *p* < 0.05 with Tukey’s post-hoc test. Various letters show significant differences between values of genotypes Kordia and Regina in six BBCHs in observed years. The Y-axes show the measured parameters, and the X-axes show the phenological phases (BBCH65-BBCH92) for both genotypes.

Regarding total flavonoid content, a significant difference between the studied genotypes was observed only at BBCH 71 during 2021 [Figure 3A; F(5, 66) = 5.372; *p* < 0.01]. However, TFC varied visibly across harvest terms. In both Kordia and Regina, flavonoid levels showed an increasing trend at the beginning and end of the season, with a decrease in July. Nonetheless, according to the statistical analysis, the increase was significant for both genotypes in the last phenological stage. The leaves of Kordia contained more flavonoids in BBCH 75–92 again.

In the second year [Figure 3B; F(5, 66) = 3.9531; *p* < 0.01], there was no difference between these two genotypes again, but distinct content was measured in the leaves between various phenological stages. The amount of flavonoids in leaves was rather higher in the second half of the harvest terms again.

#### 3.3.3. Phenolic Acids Content

Total content of phenolic acid was similar to flavonoids for Kordia and Regina in 2021 [Figure 3C; F(5, 66) = 12.489; *p* < 0.01]. However, Regina had significantly higher PAC in BBCH 71 than Kordia, but the values of these metabolites decreased again; Kordia contained more phenolic acids for the rest of sampling in this year.

In 2022 (Figure 3D), PAC was increased in BBCH 75 and BBCH 92 in both genotypes in comparison with rest of phenological stages, but there were no significant differences between Kordia and Regina as well as terms of harvesting [F(5, 66) = 1.0314; *p* = 0.40662]. In comparison with previous results, PAC in both genotypes gradually increased from BBCH 85 to BBCH 92.

#### 3.3.4. Total Antioxidant Capacity

**Figure 4:**
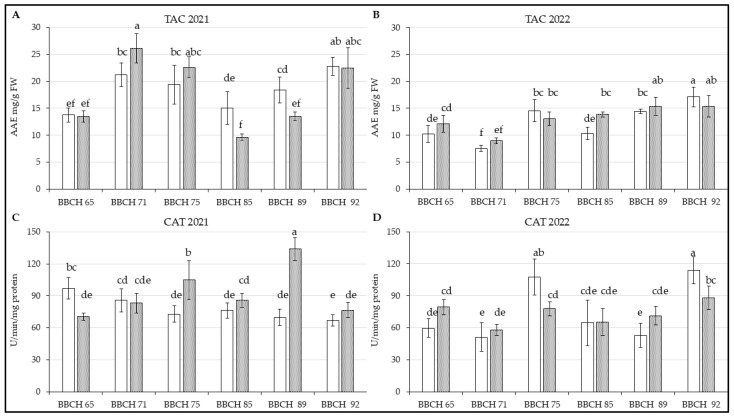
Content of measured parameters in leaves of *P. avium* two genotypes: (**A**) TAC in 2021; (**B**) TAC in 2022; (**C**) CAT in 2021; (**D**) CAT in 2022. Results are mean of 6 values ± SD. Factorial ANOVA was made for evaluation of differences if *p* < 0.05 with Tukey’s post-hoc test. Various letters show significant differences between values of genotypes Kordia and Regina in six BBCHs in observed years. The Y-axes show the measured parameters, and the X-axes show the phenological phases (BBCH65-BBCH92) for both genotypes.

Total antioxidant capacity, estimated by phosphomolybdenum assay, was highest in the leaves of Regina in BBCH 71 in season 2021 [Figure 4A; F(5, 66) = 10.227; *p* < 0.01] and there were visible different results when compared with some other stages, as well as Kordia. However, higher TAC was estimated in Kordia from BBCH 85 to BBCH 92 again. This may correlate with the higher content of phenolic metabolites (Figure S2) and other antioxidant compounds. Notably, leaves from BBCH 89 showed higher TAC compared to the previous phenological stage, even though measured polyphenolic compounds concentrations were not significantly different.

The progress of TAC measured in leaves of Kordia was similar to PAC 2022, but there was difference in the case of Regina [Figure 4B; F(5, 66) = 6.9589; *p* < 0.01]), where the decrease in BBCH 85 had not been measured.

### 3.4. Antioxidant Enzymes

#### 3.4.1. Catalase

Regarding analysis of enzymatic activity, there is a visible difference between genotypes in comparison with secondary metabolites in 2021 [Figure 4C; F(5, 66) = 35.421; *p* < 0.01]. During the first phenological phase, higher values of CAT had been observed in leaves of Kordia, but it gradually decreased in 2021. CAT activity of Regina does not follow this progress, as there was increase in BBCH 89 between BBCH 85 and 92. In 2022, the progression of CAT activity was more similar between the two genotypes [Figure 4D; F(5, 66) = 9.648; *p* < 0.01] during specific phenological stages. However, a significant increase in activity was noted for Kordia in BBCH 75 and 92, while it was lower during the other phases.

#### 3.4.2. Superoxide Dismutase

**Figure 5:**
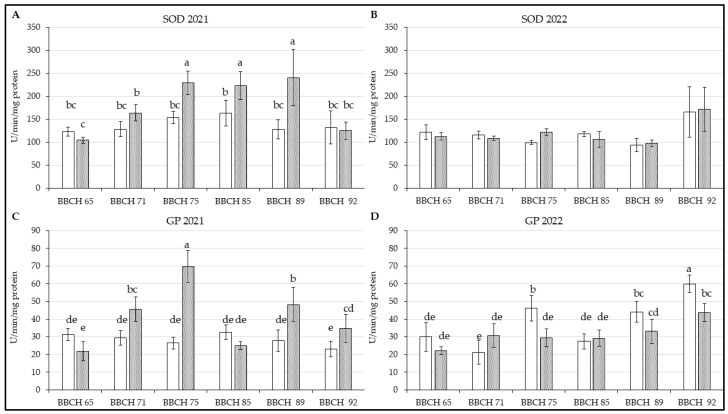
Content of measured parameters in leaves of *P. avium* two genotypes: (**A**) SOD in 2021; (**B**) SOD in 2022; (**C**) GP in 2021; (**D**) GP in 2022. Results are mean of 6 values ± SD. Factorial ANOVA was made for evaluation of differences if *p* < 0.05 with Tukey’s post-hoc test. Various letters show significant differences between values of genotypes Kordia and Regina in six BBCHs in observed years. The Y-axes show the measured parameters, and the X-axes show the phenological phases (BBCH65-BBCH92) for both genotypes.

Activity of SOD was visibly higher in leaves of Regina again, except for the first and the last phenological stage of 2021 [Figure 5A; F(5, 66) = 10.995; *p* < 0.01]. However, no significant difference were observed at these two stages. Both genotypes showed increased SOD activity from BBCH 71 to 85. The enzyme activity of the genotypes was more similar between them in the next year, as is shown in Figure 5B [F(5, 66) = 0.9958; *p* = 0.42605]. The seasonal progression differ from 2021, with SOD activity generally declining. An increase at BBCH 92, similar to that observed for CAT, was noted.

#### 3.4.3. Guaiacol Peroxidase

As Figure 5C [F(5, 66) = 36.029; *p* < 0.01] shows, GP in leaves of Kordia was again lower in comparison with the second genotype in 2021, but its progress was more stable during the season. GP of Regina was higher in most BBCHs. However, there was an observable drop in BBCH 85 as in the case of CAT and SOD, but the decrease of this GP was more prominent than these two enzymes. In 2022, the GP increased in Kordia and Regina as well [Figure 5D; F(5, 66) = 10.198; *p* < 0.01] when the latter one had more balanced progress. GP in leaves of Kordia was increased in BBCH 75 in comparison with BBCH 71 and trend of this enzyme level was generally like CAT with exception of the penultimate phenological phase. The GP of this genotype was also significantly higher in more stages than in samples from Regina.

#### 3.4.4. Ascorbate Peroxidase

**Figure 6:**
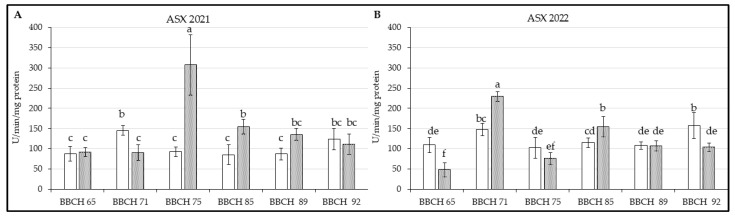
Content of measured parameters in leaves of *P. avium* two genotypes: (**A**) ASX in 2021; (**B**) ASX in 2022. Results are mean of 6 values ± SD. Factorial ANOVA was made for evaluation of differences if *p* < 0.05 with Tukey’s post-hoc test. Various letters show significant differences between values of genotypes Kordia and Regina in six BBCHs in observed years. The Y-axes show the measured parameters, and the X-axes show the phenological phases (BBCH65-BBCH92) for both genotypes.

The last estimated enzyme, ASX, had a higher level in leaves from Regina in phase BBCH 75, while Kordia had already shown an increase of this enzyme in previous term [Figure 6A; F(5, 66) = 38.759; *p* < 0.01]. GP decreased in both genotypes then, and only in the case of Kordia, its values was increased in last stage. Similar trend for this genotype was also observed in 2022 [Figure 6B; F(5, 66) = 28.857; *p* < 0.01], while ASX from leaves of Regina sweet cherry was different and the increase in BBCH 71 and BBCH 75 looked more like Kordia of this season. Nonetheless, ASX levels were higher again in the last phenological phase for Kordia.

In summary of measured results from both years, redundancy analysis (Figure S2) revealed that adjusted clarified variability is 24.86%, when the first axis explains 20.25% of total variability. Regarding environmental data, there was no direct link between temperature and rainfall, while some of the monitored parameters like PAC or CAT were strongly affected by just temperature. From Figure S2 could be also visible negative correlation between sunlight, as well as other factors, and MDA content as one of the stress indicators. The role of season (2021/2022) including its progress was manifested too.

## 4. Discussion

In recent years, climate changes driven by global warming have introduced additional factors to which plants must adapt throughout the growing season. Plants growing in situ are influenced by multiple abiotic and biotic factors that interact in various combinations, with the plant’s response depending on its genetic background. Consequently, plant reactions to elevated temperatures vary across genera, species, and ontological phases [41].

High temperatures impact anthocyanin biosynthesis in sweet cherry fruits through abscisic acid metabolism and can inhibit the photosynthetic rate in *P. avium* leaves [42]. In another study [43], elevated temperatures in *Malus domestica* led to an increase in malondialdehyde (MDA), indicative of oxidative stress and elevated hydrogen peroxide levels. This outcome contrasts with Figure S2, as higher levels of secondary metabolites and antioxidant enzymes can protect lipids from excessive damage [44]. Conversely, low nighttime temperatures negatively affected photosynthesis of post-flowering trees [45,46], with these effects persisting after repeated cold spells.

Environmental conditions during the later phases of growth were characterized by decreasing temperatures, reduced sunshine, and increased rainfall and air humidity. Senescence was observed in the leaves, marked by the degradation of photosynthetic pigments and an associated rise in flavonoid content [47], which is consistent with the observed increases in other phenolic compounds (Figure 2 and Figure 3).

Higher temperatures and light availability can be beneficial for trees, reducing the necessity to synthesize compounds with antioxidant activity. Sunshine intensity and duration directly influence flavonoid biosynthesis in plants [48,49], as seen in the total flavonoid content (TFC) for 2022 (Figure 3B) in both genotypes.

Light availability also significantly impacts the production of sweet cherry fruits and leaves, influencing traits such as chlorophyll content and net photosynthetic rate. Leaves located within the canopy center may receive insufficient light compared to peripheral leaves, potentially reducing their biosynthetic function [50]. Studies have also investigated different populations of sweet cherry leaves and their contributions to carbon transport for fruit development [51].

Genotype and rootstock, along with the spacing between cultivated trees, play a crucial role in determining plant physiology. This aligns with research [9], which reported higher photosynthetic activity, increased photosynthetic pigments, and reduced polysaccharide levels in genotypes with a more open canopy. The study also highlighted that denser canopy genotypes exhibited higher levels of phenolic compounds in their leaves, likely due to the allocation of phenylalanine towards metabolite production rather than protein synthesis. This metabolic pathway is influenced not only by light availability but also by adequate resources such as water and nutrients. As described in study [48], light intensity can regulate the activity of phenylalanine lyase, a key enzyme in plant polyphenol biosynthesis, as well as the production of phenolic acids, flavonoids, and their subclasses. Thus, the lower metabolite levels observed in 2022 (Figure 3B,D) may be partially attributed to increased sunlight compared to the previous year.

Rainfall and air humidity also positively impact phenolic content in plants [52]. For instance, *Licania macrophylla* Benth., a member of the Rosaceae family, showed a correlation between total phenolic content (TPC) and these environmental factors. During waterlogging events, the biosynthesis of antioxidant compounds may act as a protective mechanism, as observed in 2021 (Figure 2C). Elevated rainfall and air humidity, leading to higher soil moisture, significantly influenced the production of redox reactants [53]. Sweet cherry (*Prunus avium*) is particularly sensitive to root zone hypoxia [54], with more susceptible genotypes showing higher malondialdehyde (MDA) levels after prolonged soil waterlogging [55]. A similar trend was reported in another study [56], where the sweet cherry genotype ‘Burlat’ was grafted onto Gisela 5 and 6 rootstocks.

Waterlogging can damage plant tissues due to root anoxia and the accumulation of metabolites from anaerobic metabolism. Different *Prunus* species exhibit varying levels of tolerance to this type of stress [57]. Similarly, rootstocks used in sweet cherry grafting respond differently to short-term waterlogging [58]. For instance, *P. cerasus* × *P. canescens* exhibited lower malondialdehyde (MDA) levels compared to *Prunus mahaleb* and *Prunus pseudocerasus*, accompanied by increased activity of antioxidant enzymes such as catalase (CAT), glutathione reductase, and peroxidase. Conversely, higher humidity has been shown to positively affect plant growth by promoting stomatal opening and stabilizing the photosynthetic process [59]. In 2022, when environmental conditions were warmer and drier (Figure 1C,D), the content of most analyzed metabolites was slightly lower, with minimal fluctuations, particularly during the latter half of the selected phenological phases (Figure 3).

In addition to examining the effect of water on primary and secondary metabolites in various sweet cherry organs (fruits, buds, and leaves), the potential impact of rootstock was also investigated [3]. The authors utilized the Regina genotype, as in this study, grafted onto Gisela 5 or Weiroot 72, and compared their results over two years. However, sampling was conducted only once in June. Their findings indicated that plants without irrigation exhibited increased levels of hydroxycinnamic acids, total flavonols, and flavanols. Specifically, the leaves of Regina grafted onto Gisela 5 rootstock contained higher concentrations of these secondary metabolites. Given that both genotypes were grafted onto the same rootstock, this potential effect is considered negligible from this perspective.

An in vitro experiment conducted on Gisela 5 rootstock [60], where water stress was induced by increasing concentrations of PEG 6000, demonstrated that, in addition to MDA, the activity of enzymes such as CAT, SOD, and ASX was also elevated. This aligns with some of the measured results, where elevated MDA levels were accompanied by increased enzyme activity (Figure 2, Figure 4, Figure 5 and Figure 6).

As discussed by other authors [21], soil management can also influence the content of secondary metabolites and enzymes with antioxidant activity. Their study on the sweet cherry genotype Dilema, grafted onto Antipka rootstock, involved treating the soil around the observed trees with pure steam, manual weeding, or covering it with mowed grass. This management approach was also employed at RBFI Holovousy. The authors observed biochemical changes in the leaves across four phases, with most analyzed parameters gradually increasing during tree development from 2017 to 2019. However, there were differences between individual years, as described here (Figure 2 and Figure 3).

Their results [21] also demonstrated that elevated MDA content was accompanied by higher levels of phenolics and antioxidant enzymes. The fluctuations observed in Kordia and Regina could be attributed to the potential role of phytohormones released from mulching plants [61,62], which improve the environment for soil microorganisms [63]. This effect can manifest in both leaves and fruits [20]. The authors also suggested that the decrease in observed metabolites and enzyme activity could be influenced by prolonged drought, potentially explaining the lower values in 2022 (Figure 4D and Figure 5D), when precipitation levels were reduced compared to the previous year.

However, other factors could also play a role. For instance, in the case of phenolic acids, the difference between the studied variants was not significant in one year. The authors proposed that the absence of fruits in that season could affect metabolite levels, although both Kordia and Regina had normal production in both observed years. A similar result was observed in the Vanda genotype [12].

As mentioned in several cases above, rootstock plays a crucial role in response to environmental stress. Several rootstocks from the *Prunus* family were tested in pots against waterlogging, and tolerant specimens retained physiological functions (gas exchange, photosynthesis) and pigment content (chlorophyll, carotenoids), which contribute to antioxidant protection [55]. Leaves of the tolerant genotype contained similar MDA levels, while in the susceptible genotype, MDA levels gradually increased during the experimental period.

Different rootstocks for sweet cherry may also exhibit varying sensitivity to soil aeration and flooding, impacting their vegetative growth. However, overall tolerance should be evaluated in conjunction with the grafted genotype [64]. Variations in secondary metabolite content could also be explained by higher metabolic activity during leaf development, as described for young lingonberry leaves [65]. They also discussed several factors, such as light, drought, and plant location, which contribute to increased biosynthesis of various compounds.

Regarding the genus *Prunus*, other factors such as seasonality and genotype have been reported to influence the variability of phenolic content and antioxidant activity in *Prunus amygdalus* L. [66]. Similar variations were observed in the leaves of sugar maple (*Acer saccharum* Marshall), which exhibited higher phenolic content in May and June, and again in October [67]. Our data align with other studies [68], where the content of chlorogenic acid and rutin was measured in the leaves of the *P. avium* genotype Lapins grafted onto three different rootstocks, showing a similar trend in metabolite content throughout the season.

Conversely, *P. avium* cv. Lapins on the Santa Lucia 64 rootstock exhibited increased membrane damage between BBCH stages 77 and 86 [23]. Although the stages and genotypes differ, they are relatively close to the observed phases in this study, with a similar increase noted for both genotypes at BBCH stages 75 and 85 in 2022 (Figure 2B). Additionally, the authors [23] recorded significantly higher total phenolic content in the second measurement, while the rise in antioxidant activity was smaller and negligible. Nonetheless, the trend regarding membrane damage and TPC is more consistent with other observed phenological phases in 2021 and 2022.

Recent research has examined the content of chlorogenic acid, an ester of caffeic and quinic acid, apigenin-7-O-glucoside, and antioxidant activity in the leaves of *Cirsium vulgare* (Savi) Ten. across various phenological phases, noting seasonal fluctuations [69]. Although *C. vulgare* is neither from the same family nor a tree, their research demonstrated that the biosynthesis of phenolic compounds can vary across different developmental stages and years, with phases of highs, lows, or relatively stable content. This is consistent with findings from other authors who described variations in flavonoid content in various edible plants, influenced by seasons, genotype, and growth conditions [70]. The presence of compounds contributing to total metabolite content is also variable, potentially explaining differences between phenological phases and genotypes [71].

Regarding sweet cherry fruits, the phenolic compound content was compared in the same genotypes [72] as in this study. The authors did not observe significant differences between 2015 and 2016; however, their results could be influenced by factors such as different climatic conditions, experimental setups in different seasons, and a focus on fruits.

A recent study [73] described higher flavonol content, such as quercetin and its glycoside rutin, in the leaves of birches (*Betula pendula* L.). The hypothesis that these metabolites protect against aphids was not proven. However, the authors suggest that these and other polyphenolics may act as carbon sinks in senescing leaves, defending against light, including UV radiation, and other stress types. Flavonols, along with other defense mechanisms, protect cell structures and processes by eliminating ROS, although their amount may not be sufficient in older leaves. This could explain the higher TFC in 2021 (Figure 3A); however, the senescence process can vary among species, with flavonol biosynthesis related to higher chlorophyll degradation [47].

As mentioned in the introduction, other parts of *P. avium*, besides the fruits, could be valuable sources of compounds with beneficial biological activity. Higher values of ascorbic acid, total carotenoids, and TPC were measured in leaves compared to fruits, with a similarly increased antioxidant capacity [19]. Kordia and Regina again appeared as genotypes with higher TPC.

In addition to their effects in leaves, flavonoids and other phenolic compounds may also play a role in the oxidation-reduction reactions occurring in the buds of *P. avium*, contributing to the ontogenesis of these plant parts [74].

Higher TPC content was measured in water extracts from dried leaves of sweet cherry of unspecified genotype, along with antioxidant assays like DPPH, ABTS, and the phosphomolybdenum method/TAC [11], with trends consistent with the results in Figure S2. The increased TFC in that study was estimated primarily in methanol, using rutin as a standard.

## 5. Conclusions

A two-year observation of selected biochemical parameters in the leaves of *P. avium* Kordia and Regina indicated that the content of secondary metabolites is primarily dependent on temperature, although other climatic factors may also play a role. Additionally, the phenological stage appears to be significant, particularly for the utilization of leaves for their bioactive compounds. Regarding the potential resistance of Kordia and Regina to environmental conditions, both genotypes exhibited similar trends during the selected seasons. This study could be extended to include a more detailed analysis of changes in secondary metabolite content in sweet cherry leaves across various phenological phases.

## Data Availability

Data is contained in the article.

## Supplementary Materials

The following supporting information can be downloaded at: https://www.mdpi.com/article/10.3390/life14121567/s1, Table S1: Phenological phasesKlikněte nebo klepněte sem a zadejte text. [28]. Figure S1: Calibration curves of standards for estimation of selected biochemicals parameters. Figure S2: Redundancy analysis (RDA) with supplementary variables (genotypes and years).
