# Supplementary material for: Changes in Secondary Metabolites Content and Antioxidant Enzymes Activity in Leaves of Two Prunus avium L. Genotypes During Various Phenological Phases
Source: Life (Basel). 2024 Nov 29;14(12):1567. doi: 10.3390/life14121567 (PMC11678347; doi:10.3390/life14121567)
Supplement: Supplementary file 1 [file life-14-01567-s001.zip › life-3317901-supplementary.pdf]

Supplementary materials

Table S1. Phenological phases [28].

No. of phase	Short description	Year	
		2021	2022
BBCH 65	Full flower, 50 % of flowers are opened or 50 % plants flowering	19.5.	13.5.
BBCH 71	10% of fruits in full size or fruit reached 10 % of full size	17.6.	27.5
BBCH 75	50% of fruits in full size or fruit reached 50 % of full size	01.7.	13.6.
BBCH 85	Progressive ripening or typical coloring of species/genotype	16.7.	30.6.
BBCH 89	Full ripening, fruits are fully and typical colored, fruit dropping	28.7.	15.7
BBCH 92	Leaves change color or falling	23.9.	13.9.

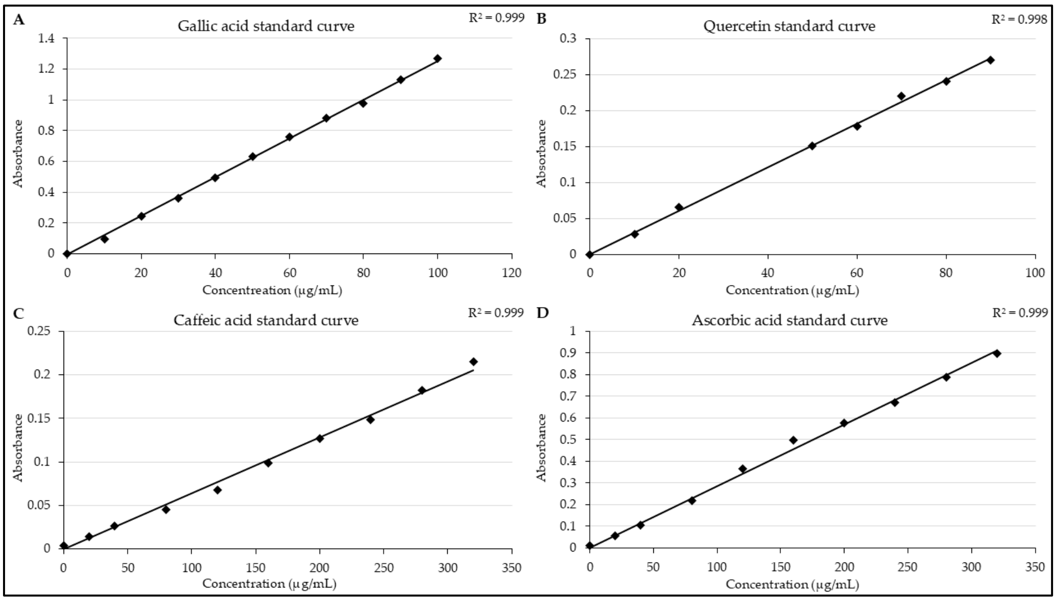

Figure S1. Calibration curves of standards for estimation of selected biochemicals parameters: (**A**) Gallic acid standard curve for total phenolic content (TPC); (**B**) Quercetin standard curve for total flavonoid content (TFC); (**C**) Caffeic acid standard curve for phenolic acids content (PAC); (**D**) Ascorbic acid standard curve for total antioxidant capacity (TAC).

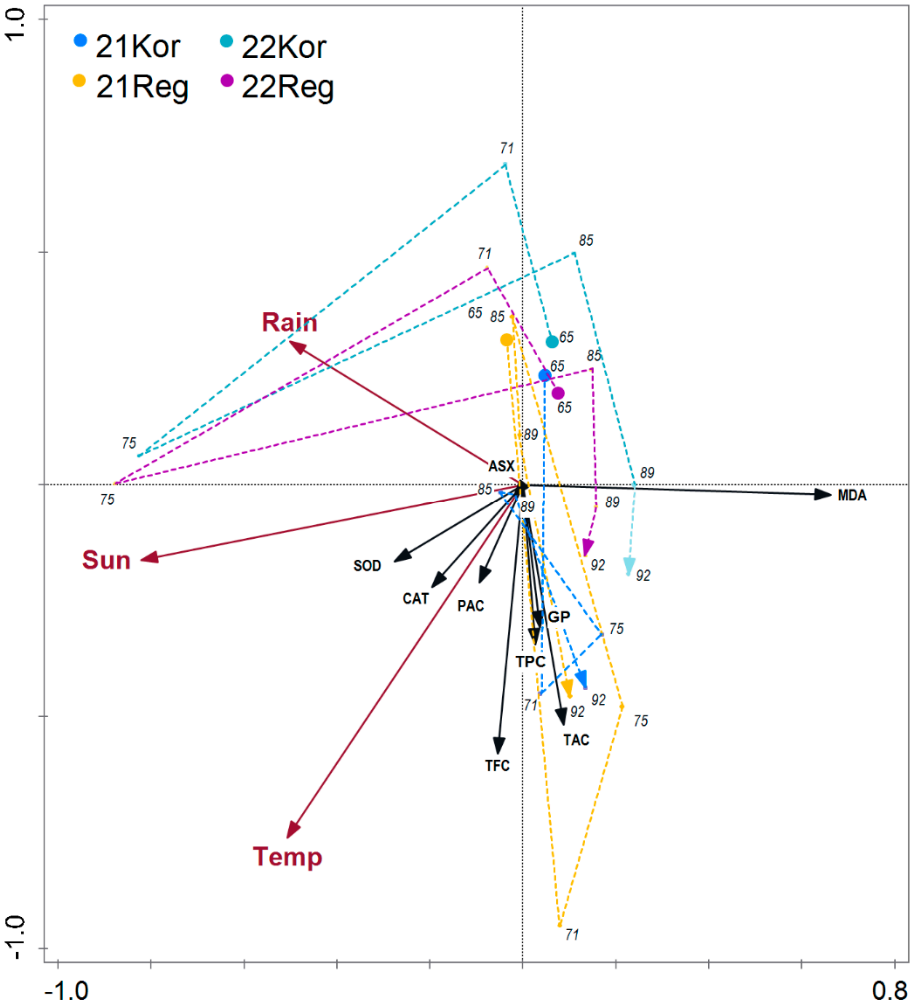

Figure S2. Redundancy analysis (RDA) with supplementary variables (genotypes and years). Total explanatory variables account for 26,31%. The first two ordination axis included 26,12% of the total data variability. The dark red arrows indicate the explanatory variables: precipitation (Rain), sunshine (Sun), and temperature (Temp). The supplementary variables are represented by 21Kor (the first year for the Kordia genotype), 22Kor (the second year for the Kordia genotype), 21Reg (the first year for the Regina genotype), and 22Reg (the second year for the Regina genotype). The numbers correspond to the different phenological phases. The black arrows indicate the measured parameters.
